# CD4^+^ T cells related to disease severity in elderly and frailty community‐acquired pneumonia patients: A retrospective cohort study

**DOI:** 10.1002/iid3.1009

**Published:** 2023-09-29

**Authors:** Jue Wang, Lu Pei, Ting Zhao, Xiaoli Liu, Quanrong Wang, Shiyu Zhang, Jubo Li, Hongwei Wu, Dongsheng Niu

**Affiliations:** ^1^ Jincheng People's Hospital Jincheng China; ^2^ Jincheng Hospital Affiliated to Changzhi Medical College Jincheng China; ^3^ Critical Medicine Department of Huangpi District People's Hospital Affiliated to Jianghan University Wuhan China

**Keywords:** CD4^+^ T cells, elderly patients, frailty patients, PCT, severe community‐acquired pneumonia (SCAP)

## Abstract

**Backgrounds:**

Elderly and frailty individuals show a more senescent immune system, which may relate to worse outcome in community‐acquired pneumonia (CAP). This study aimed to explore prognostic factors related to immune.

**Methods:**

Sixty of elderly (≥65 years) and frailty (clinical frailty scale ≥5 scores) nonsevere CAP patients and 60 severe CAP (SCAP) patients were recruited at our center. Clinical and laboratory data, and several assessment scores were collected.

**Results:**

Compared with nonsevere CAP group, the elderly and frailty SCAP patients showed higher level of BMI, PaCO2 and lactate in arterial blood‐gas, CURB‐65 score, ICU admission, mechanical ventilation, shock accidence, and longer hospital stay using two‐tailed *t* test. The SCAP group also showed increased CRP, IL‐6, and PCT, and decreased CD3^+^ T cells, CD4^+^ T cells, and CD8^+^ T cells. Logistic regression analysis showed that CD4^+^ T cells, IL‐6 and PCT were independent prognostic factors for SCAP. The area under the receiver operating characteristic (ROC) curve for CD4^+^ T cells combined with PCT was 0.771 (95% CI 0.683–0.859), and the sensitivity and specificity were both 76.7%. Paired *t* test analysis showed that low CD4^+^ T cells in SCAP patients increased after treatment.

**Conclusions:**

CD4^+^ T cells decreased in elderly and frailty SCAP patients, and CD4^+^ T cells combined with PCT were relatively accurate in the prediction of elderly and frailty SCAP.

## Graphical abstract

CD4^+^ T cells decrease in elderly and frailty SCAP patients, which indicates a worse outcome. CD4^+^ T cells combined with PCT are powerful prognostic biomarkers in evaluating the severity of community‐acquired pneumonia.

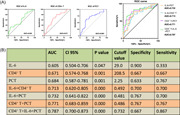

## 1 INTRODUCTION

Community‐acquired pneumonia (CAP) is one of the leading infectious causes of hospitalization, morbidity, and death globally.^1^ If not successfully treated, respiratory failure and shock may arise, thus CAP progress to severe community‐acquired pneumonia (SCAP), which is characterized by high morbidity and mortality. For reasons not completely clear, elderly and frailty CAP patients are particular at risk of developing SCAP.^2^ Considering to the aging of world's population, the management of SCAP in elderly patients becomes more challenging. Initial identification of SCAP in elderly is important for instituting different management and monitoring measures.

In pneumonia, the host response has been evaluated from various perspectives, mainly focused on inflammatory response and innate immunity. Besides of having more chronic comorbidities, elderly individuals possess impaired immunity due to age‐associated changes compared to younger adults.^3^ Lymphopenia has been identified as an independent risk factor in CAP,^4^ which reminds us the role of adaptive immunity. Age‐related defects of T cells in immunosenescent modifications have been described. The T cells proportion at different stages change, with peripheral naïve T cells reduce and senescent‐like T cells elevate.^5^ TCR repertoire decrease, which results in weak immune response to pathogens.^6^, ^7^ Several studies have been conducted in elderly patients, and found the relationship between CAP and various subtype T cells, such as CD4^+^ T cells and Treg cells. In a recent study, elderly SCAP patients showed lower CD4^+^ T cells in survival group than that of mortality group.^8^

Frailty develops as a consequence of age‐related decline in physiological function, and relates to poor prognosis under minor stressor events.^9^ Immunosenescent modifications affect the entire immune system, including innate and adaptive immunity. Clinical frailty scale (CFS) is widely used with values ranging from 1 to 9, and ≥5 is thought to be frailty. CD4^+^ T cells are found related to elderly CAP patients, while there are little research focusing on the topic of CD4^+^ T cells in elderly and frailty CAP patients.

We aim to characterize the clinical and laboratory features (including inflammatory markers and T cell subsets) in elderly and frailty nonsevere CAP and SCAP patients, and to explore the risk factors for SCAP, thus helping to optimize the management of SCAP patients.

## 2 METHODS

### 2.1 Study design and patients

This study was conducted in Jincheng People's Hospital Affiliated to Shanxi Medical University. Community‐acquired pneumonia patients aged ≥65‐year‐old were enrolled in the period from between July 2020 and July 2022. This study was a retrospective cohort study.

### 2.2 Ethical statement

The hospital's ethics committee approved this study (No.20200319001). All participants provided informed consent when they registered for the current study.

### 2.3 Inclusion and exclusion criteria

The inclusion criteria were as follows: patients aged ≥65 years old with complete medical records; patients satisfying the diagnostic criteria of CAP and SCAP; clinical frailty scale ≥5 scores (CFS scores of 5–9 indicate that the patients need everyday help); hospitalization within 48 h of symptom onset.

The exclusion criteria were end‐of‐life clinical status, HIV positivity, oncology, hematology, transplant recipients, COVID‐19 infection, received ≥20 mg/d of steroid in last 3 months, received chemotherapy or radiation therapy during the last 6 months.

### 2.4 Grouping

The patients in our study were divided into two groups: Nonsevere CAP group and SCAP group. CAP and SCAP were diagnosed according to the guideline by the Chinese Medical Association.^10^ CAP was diagnosed as the presence of a new or progressive patchy infiltrates or solid lobar changes on chest X‐ray or computed tomography, plus acute respiratory symptoms and/or signs (fever, chills, cough, expectoration, chest pain, dyspnea). SCAP was defined if the CAP patient met at least one of the following criteria: required tracheal intubation and mechanical ventilation, experienced shock and still needed vasoactive drugs after adequate fluid resuscitation; or at least three of minor criteria: respiratory rate ≥30 bpm, oxygenation index ≤250 mmHg, infiltration in multiple lung lobes, disturbance of consciousness, and/or disorientation.

### 2.5 Data collection

We collected data on demographic characteristics, such as age, sex, height, and weight. The comorbidities were also recorded, including chronic cardiovascular disease, chronic respiratory disease, chronic kidney disease, and diabetes mellitus. Pneumonia severity index (PSI) score and confusion, urea, respiratory rate and age 65 (CURB‐65) score were used assessing the severity of illness. The clinical data including arterial blood gas, white blood cell count, hemoglobin, platelet, C‐reaction protein (CRP), procalcitonin (PCT), interleukin‐6and chest X‐ray, or CT were obtained within 24 h after admission. The first radiological examination, whether X‐ray or CT, were used for pneumonia diagnose.

### 2.6 Flow cytometry analysis

Four milliliter of peripheral venous blood was collected in a EDTA tube. Erythrocytes were lysed and supernant was removed. The samples were stained with CD45‐FITC, CD3‐PE, CD4‐APC, CD8‐PerCP/Cy5.5 (Beckman Coulter), and incubated for 30 min at 4°C. The cells were washed twice and diluted with 1X PBS. The samples were immediately detected using FACSCantoⅡ cytometer (Becton Dickinson), and analyzed with by Flowjo software (Treestar).

### 2.7 Statistical analysis

Statistical analysis was performed using IBM SPSS Version 20.0 software (IBM). Continuous data were expressed as mean ± standard deviation. The two‐tailed Student's *t* test was used for comparisons between two groups. Categorical variable results were expressed as number (%) and compared with the *χ* ^2^ test. Correlations were assessed using the Spearman test. Logistic regression analysis determined the independent risk factors for SCAP. The receiver operating characteristic (ROC) curve was performed, and the area under the curve (AUC) was calculated to predict the ability of risk factors to predict SCAP. Changes before and after pneumonia treatments were evaluated with paired *t* test. *p* < .05 was considered statistically significant.

## 3 RESULTS

### 3.1 General characteristics

A total of 156 patients met inclusion criteria, and 36 patients met the exclusion criteria (combined severe heart failure, renal failure, ≥20 mg/d steroid administration, received chemotherapy or radiation therapy, etc). The Nonsever CAP group included 60 patients, and SCAP group included 60 patients eventually. Demographic data of the two groups were shown in Table 1. The age, clinical frailty scale, comorbidities and PSI score were comparable between the two groups. SCAP group showed higher BMI (*p* < .05) than those in nonsevere CAP patients, as well as more severity assessed by CURB‐65 score (*p* < .01). Besides, the hospital stay (*p* ＜ .01), ICU admission proportion (*p* ＜ .01), mechanical ventilation (*p* ＜ .01) and shock accidence (*p* ＜ .01) were significantly higher in SCAP group than those of nonsevere CAP group.

**Table 1:** The clinical characteristics of the studied groups.

	Nonsevere CAP	Severe CAP	*p* Value
Gender, male *n* (%)	28 (46.7)	30 (50)	.715
Age, year	73.1 ± 4.4	73.9 ± 4.4	.319
Clinical frailty scale	5.83 ± 0.977	6.1 ± 0.986	.139
BMI	23.95 ± 2.05	24.96 ± 2.63	.015
Co‐morbidies, *n* (%)			
Chronic cardiovascular disease	24 (40)	32 (53.3)	.143
Chronic respiratory disease	28 (46.7)	30 (50)	.715
Chronic kidney disease	14 (23.3)	18 (30)	.409
Diabetes mellitus	24 (40)	26 (43.3)	.711
Severity Index			
Pneumonia Severity Index score	123 ± 11	120 ± 12	.082
CURB 65 score	2.0 ± 0.69	2.63 ± 0.84	.000
Hospital stay (day)	8.5 ± 1.4	15.7 ± 2.4	.000
ICU admission	6 (10)	22 (36.7)	.001
Mechanical ventilation	8 (13.3)	26 (43.3)	.000
Shock	2 (3.3)	14 (23.3)	.001

*Note*: The two‐tailed Student's *t* test was used for comparisons between two groups. Categorical variable results were expressed as number (%) and compared with the *χ* ^2^ test.

Abbreviations: BMI, body mass index; CURB 65, confusion, urea, respiratory rate and age 65; ICU, intensive care unit; WBC, white blood cell.

### 3.2 Inflammatory and T cells immune findings between the two groups

**Figure 1:**
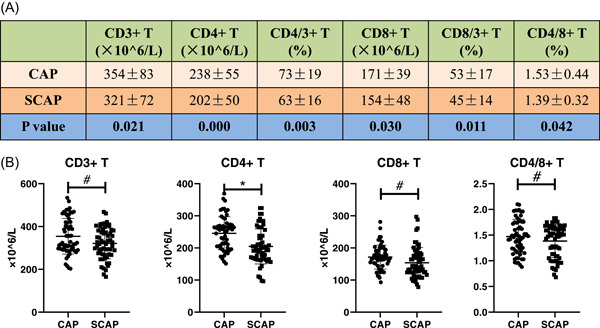
Inflammatory markers and T cells between CAP and SCAP group. (A and B) The CD3^+^ T cells, CD4^+^ T cells, CD8^+^ T cells, and ratio of CD4^+^/CD8^+^ T cells were decreased in elderly and frailty SCAP group. *N* = 60. ^#^ *p* ≤ .05, and *p ≤ .01. The two‐tailed Student's *t* test was used for comparisons between two groups. Categorical variable results were expressed as number (%) and compared with the *χ* ^2^ test.

As shown in Table 2, the pH value, PO2/FiO2, white blood cell, neutrophils, lymphocytes, alanine transaminase (ALT), aspartate transaminase (AST), albumin, estimated glomerular filtration rate (eGFR), hemoglobin, and platelet were comparable between the two groups. SCAP group showed higher BMI (*p* ＜ .05), PaCO2 (*p* ＜ .05) and lactate (*p* ＜ .01) than those in CAP patients. The concentration of serous C‐reaction (*p* ＜ .05), PCT (*p* ＜ .05), and IL‐6 (*p* ＜ .01) were significantly higher in SCAP patients than those in the compared group. As shown in Figure 1, CD3^+^ T lymphocytes (*p* ＜ .05), CD4^+^ T lymphocytes (*p* ＜ .01), CD8^+^ T lymphocytes (*p* ＜ .05), and CD4^+^/CD8^+^ T lymphocyte ratio (*p* ＜ .05) were all decreased in the SCAP group, compared with the nonsevere CAP group.

**Table 2:** The clinical tests of the studied groups.

	Nonsevere CAP	Severe CAP	*p* Value
Arterial blood gase			
pH, median (IQR)	7.42 ± 0.04	7.40 ± 0.07	.065
PaO2/FiO2	394.9 ± 34.7	390.3 ± 50.8	.564
PaCO2, mmHg	37.8 ± 4.3	40.4 ± 6.6	.012
Lactate	1.50 ± 0.70	2.27 ± 1.05	.000
Routine blood			
White blood cells, ×10^9	12.96 ± 2.25	13.4 ± 3.03	.376
Neutrophils, ×10^9	10.84 ± 2.02	11.09 ± 3.14	.607
Lymphocytes, ×10^9	1.00 ± 0.18	0.95 ± 0.18	.099
Hemoglobin, g/L	111.4 ± 11.3	112.0 ± 9.5	.727
Platelet, ×10^9	294 ± 39	294 ± 54	.067
Inflammatory marker			
C‐reaction protein, mg/L	40.1 ± 13.7	46.5 ± 19.4	.039
Procalcitonin, ug/L	1.92 ± 1.83	2.84 ± 2.15	.013
Interleukin‐6, ug/L	36.3 ± 9.7	41.9 ± 12.8	.007
Biochemistry			
ALT, U/L	42.4 ± 15.4	39.4 ± 18.3	.333
AST, U/L	34.7 ± 7.0	36.0 ± 12.3	.478
Albumin, g/L	35.4 ± 6.5	34.2 ± 6.9	.306
eGFR, mL/(min × 1.73 m^2^)	94.9 ± 22.0	91.5 ± 23.4	.419
Radiography			
Chest X‐ray, *n*	18	15	
Chest CT, *n*	42	45	

*Note*: The two‐tailed Student's *t* test was used for comparisons between two groups.

Abbreviations: ALT, alanine transaminase; AST, aspartate transaminase; eGFR, estimated glomerular filtration rate.

### 3.3 CD4^+^ T cells, IL‐6, and PCT are independent risk factors for SCAP

**Figure 2:**
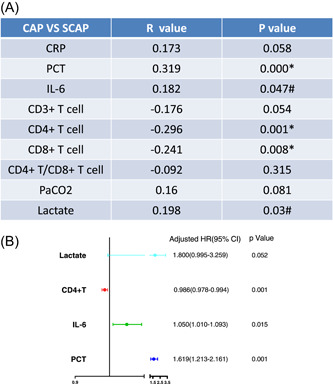
CD4^+^ T cells, IL‐6, and PCT were independent risk factors for SCAP. (A) PCT, IL‐6, CD4^+^ T cells, CD8^+^ T cells, and lactate were related to the elderly and frailty SCAP group. (B) Logistic analysis showed that CD4^+^ T cells, PCT, and IL‐6 were independent risk factors for elderly and frailty SCAP. *N* = 60. ^#^ *p* ≤ .05, and *p ≤ .01. Correlations were assessed using the Spearman test. Logistic regression analysis determined the independent risk factors for SCAP. CRP, C‐reactive protein; IL‐6, interleukin‐6; PCT, procalcitonin.

In correlation analysis, we found that PCT (*R* .319, *p* = .000), IL‐6 (*R* .182, *p* = .047), CD4^+^ T cells (*R* −.296, *p* = .001), CD8^+^ T cells (*R* −.241, *p* = .008) and lactate (*R* .198, *p* = .03) were correlated with SCAP (Figure 2A). Then, results of logistic regression were shown in Figure 2B, CD4^+^ T cells (HR 0.986, 95% CI 0.978–0.994, *p* = .001), IL‐6 (HR 1.05, 95% CI 1.01–1.093, *p* = .015) and PCT (HR 1.619. 95% CI 1.213–2.161, *p* = .001) were independent risk factors for SCAP.

### 3.4 CD4^+^ T cells combined with PCT are powerful prognostic biomarkers for SCAP

**Figure 3:**
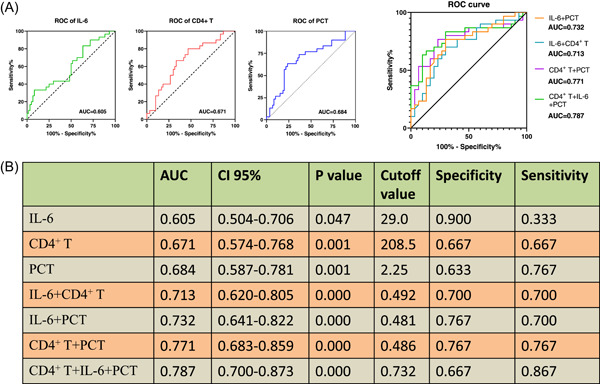
ROC analysis of individual variable and multiple variables in SCAP. (A and B) The AUC of IL‐6, CD4^+^ T cells and PCT were 0.605, 0.671, and 0.684 respectively in predicting SCAP. (B and C) The AUC of IL‐6 +CD4^+^ T cells, IL‐6 +PCT, CD4^+^ T cells + PCT, IL‐6 +CD4^+^ T cells +PCT were 0.713, 0.732, 0.771, and 0.787 respectively in predicting SCAP. *N* = 60. ^#^ *p* ≤ .05, and *p ≤ .01. The receiver operating characteristic curve was performed. AUC, area under curve; CRP, C‐reactive protein; IL‐6, interleukin‐6; PCT, procalcitonin.

In the ROC curve analysis, the AUC of IL‐6, CD4^+^ T cells and PCT were 0.605 (95% CI 0.504–0.706, *p* = .047), 0.671 (95% CI 0.574–0.768, *p* = .001) and 0.684 (95% CI 0.587–0.781, *p* = .001) respectively in predicting SCAP (Figure 3A,B). Compared to single biomarkers, combined markers showed better predictive effects (Figure 3B,C). The AUC of IL‐6 +CD4^+^ T cells, IL‐6 + PCT, CD4^+^ T cells +PCT, IL‐6 + CD4^+^ T cells +PCT were 0.713 (95% CI 0.62–0.805, *p* = .000), 0.732 (95% CI 0.641–0.822, *p* = .000), 0.771 (95% CI 0.683–0.859, *p* = .000), and 0.787 (95% CI 0.7–0.873, *p* = .000) respectively in predicting SCAP. At the same time, the positive and negative predictive values were both 0.767 in CD4^+^ T cells +PCT model.

### 3.5 CD4^+^ T cells, IL‐6, and PCT restored after treatment in SCAP patients

**Figure 4:**
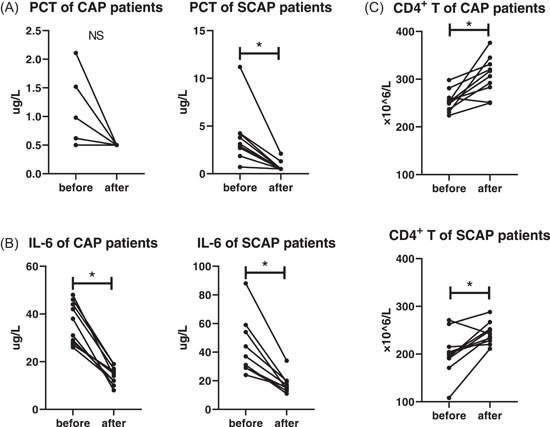
CD4^+^ T cells, IL‐6, and PCT were restored after treatment of SCAP patients. (A–C) IL‐6 decreased and CD4^+^ T cells increased in CAP group after treatment, with not significantly decreased PCT. (A–C) In SCAP group, treatment descended the concentration of PCT and IL‐6, and elevated CD4^+^ T cells. *N* = 10. ^#^ *p* ≤ .05, and *p ≤ .01. Changes before and after pneumonia treatments were evaluated with paired *t* test. IL‐6, interleukin‐6; PCT, procalcitonin.

Among the 120 enrolled patients, 10 of nonsevere CAP and 10 SCAP patients were randomly included. Data before and after pneumonia treatment were compared. After treatment, IL‐6 (*p* ＜ .01) decreased and CD4^+^ T cells (*p* ＜ .01) increased in nonsevere CAP group, with not significantly decreased PCT (Figure 4A–C). In SCAP group, treatment also reduced the concentration of PCT (*p* ＜ .01) and IL‐6 (*p* ＜ .01), and elevated CD4^+^ T cells (*p* ＜ .01) (Figure 4A–C).

## 4 DISCUSSION

This study retrospectively analyzed the risk factors for senior and frailty SCAP, and explored the predictive effect of CD4^+^ T cells on SCAP. Apart from the markers reflecting pulmonary function (arterial blood gas) and inflammation (leukocyte, CRP, and PCT), comorbidities of major organs (heart, kidney, diabetes, and lung), which might influence the outcome of CAP, and pneumonia severity scoring system (PSI and CURB‐65) were included in our research. The results revealed that: (1) the SCAP group had increased lactate and PaCO2, CURB‐65 score, CRP, IL‐6 and PCT, and decreased CD3^+^ T cells, CD4^+^ T cells and CD8^+^ T cells. (2) Logistic regression analysis showed that CD4^+^ T cells, IL‐6, and PCT were independent prognostic factors for SCAP. (3) The area under the receiver operating characteristic (ROC) curve for CD4^+^ T cells combined with PCT was 0.771 (95% CI 0.683–0.859), and the sensitivity and specificity were both 76.7%. (4) Low CD4^+^ T cells increased after treatment in SCAP patients. Overall, we found that CD4^+^ T cells decreased in elderly and frailty SCAP patients, and CD4^+^ T cells combined with PCT were relatively accurate in the prediction of elderly and frailty SCAP.

With the improvement of medical conditions and life expectancy, group of elderly and frailty persons rapid expand in the developed and developing counties. SCAP is associated with substantial morbidity and mortality, especially in elderly and frailty individuals. The infection fatality ratio also elevates as increase in age in the COVID‐19 pandemic, especially high in elderly and frailty patients.^11^ The elderly and frailty individuals suffer various health issues, such as immunosenescent, innutrition, comorbidities, and antibiotic resistance, thus preferring to a poor prognosis in CAP.^12^, ^13^, ^14^ Hence, the elderly and frailty patients should be paid separately attention in SCAP studies.

In our study, we found increased PaCO2 and lactate expression in arteria blood‐gas of SCAP group, and lactate was related to SCAP. Lactate level is independently associated with lower mortality in CAP induced sepsis patients.^15^ Frenzen et al.^16^ reported that lactate had independent predictive value in CURB‐65 score of CAP patients. A clinical trial showed that a model including lactate and CURB‐65 was superior to CURB‐65 alone in predicting severity and prognosis in CAP patients.^17^ Studies have showed that adequate fluid resuscitation guided by lactate would improve outcome in critically patients, including SCAP.^18^ Our study did not find the predictive value of lactate in distinguish SCAP from CAP patients, which might lie on the difference between the severity of CAP in our and others' research.

Our study found higher CURB‐65 score, not PSI score in SCAP group. PSI and CURB‐65 are scoring systems widely used in predicting pneumonia severity, especially in elderly patients,^19^, ^20^ while both of them are less useful in identifying SCAP requiring ICU admission.^21^ Different from the complex of PSI score, CURB‐65 is simple, and widely recommended in predicting severity of CAP patients for its accuracy. CURB‐65 score assigns less importance to the impact of comorbidities, which are comparable between CAP and SCAP group in our study, thus may clarify our results. Consistent with our study, CURB‐65, though easy to use, is reported neither sensitive nor specific for predicting mortality in CAP patients.^22^

Over the past few years, numerous studies have focused on the significance of single biomarkers for SCAP. Our results showed that the SCAP patients had higher levels of CRP, PCT, and IL‐6 than those in CAP patients, which had been thought as traditional biomarkers.^23^, ^24^ Besides, our study suggested that PCT performed best as a single indicator in distinguishing SCAP from CAP. Consistence with our result, Liu et al.^25^ reported that PCT was strongly related to the severity of pneumonia complicated by sepsis. Similar findings have been reported in other studies of SCAP.^23^, ^26^

Our study found that lower CD4^+^ T cells helped to recognize SCAP patients from elder and frailty CAP patients. CD4^+^ T cells play an important role in regulating cellular and humoral immunity. Decline of immune function results in worse prognosis in pneumonia. Several studies have revealed that decreased CD4^+^ T cells indicate higher mortality. In a study enrolled 120 elderly SCAP patients, decreased CD4^+^ T cells were independent prognostic factors of mortality.^8^ Similarly, a recent study about elderly patients with pneumonia showed that lower levels of CD4^+^ T cells were observed in the early deceased group than the late deceased patients or survivors.^27^ In elderly individuals, CD4^+^ T cells tend to toward the pro‐inflammatory Th17 cells and reduce toward the Treg cells.^28^ Th17 cells in bronchoalveolar lavage and peripheral are reported relating to CAP,^29^ and impaired Treg cell response is associated with worse prognosis in elderly CAP patients.^28^ Most of SCAP combines with sepsis‐related immunosuppression, and CD4^+^ T cells decrease may due to proliferation and apoptosis.^30^ Regardless of age, Feng et al.^31^ also found lower CD3^+^ T cells, CD4^+^ T cells, lymphocyte levels and CD4^+^/CD8^+^ T cells ratio in stroke‐associated pneumonia than those in nonpneumonia stroke patients. Besides, interstitial pneumonia (induced by COVID‐19 or not), decreased memory effector T cells were also found, while T cells and CD4^+^ T cells were comparable.^32^ All of these reminded us that T cells, especially subtypes of T cells, changed in various kinds of pneumonia. T cells‐induced immune response was far from clear in the defense against bacterial or viral pneumonia. Flow cytometry has been popular in many hospitals, and various lymphocytes can be detected easily, thus may provide a guide for clinical immune support therapy.

Our study also has some limitations. First, the number of participants is small, thus affecting the validity of the results. Second, there are many other confounding factors affecting the outcomes, such as the antibiotic or intravenous immunoglobulin treatment, and our risk factors included may not be enough. Third, some of SCAP patients transferred to ICU received therapy of ICU doctors, which may different from ours, thus affecting the final prognosis. Fourth, the positive detection rate of pathogenic bacteria was relatively low in our study, and we can't include the microbiological analyses. Finally, our study is an exploratory observational study, and we don't conduct sample calculation, which might affect the validity of our results. Further investigation is warranted to verify our results.

## 5 CONCLUSION

In summary, we find that circulation CD4^+^ T cells decrease, which indicates a worse outcome in elderly and frailty SCAP patients. CD4^+^ T cells combined with PCT might be a potential prognostic biomarker in evaluating the severity of community‐acquired pneumonia. The adaptive immune reaction (including peripheral Treg and CD8+ T cells, or T cell subtypes resident in lung) are reported associating with the prognosis of SCAP. Monitoring the immune status might contribute to identify the severe pneumonia patients, and appropriate immune support therapy might help to control the disease exacerbation and improve clinical outcomes.

## AUTHOR CONTRIBUTIONS

**Jue Wang**: Writing original draft; review and editing (equal) and performing the research (lead). **Lu Pei**: Formal analysis (lead). **Ting Zhao**: Writing original draft (equal) and performing the research (supporting). **Xiaoli Liu**: Methodology (lead). **Quanrong Wang**: Review and editing (equal). **Shiyu Zhang**: Writing original draft; performing the research (supporting). **Jubo Li**: Analyzing the data, review, and editing (equal). **Hongwei Wu**: Analyzing the data (equal). **Dongsheng Niu**: Conceptualization; review and editing (lead). All authors have read and approved the final manuscript.

## CONFLICT OF INTEREST STATEMENT

The authors declare no conflict of interest.

## Data Availability

I confirm that my article contains a data availability statement even if no data is available (list of sample statements) unless my article type does not require one.
